# The influence of preconditioning with low dose of LPS on paraquat-induced neurotoxicity, microglia activation and expression of α-synuclein and synphilin-1 in the dopaminergic system

**DOI:** 10.1007/s43440-021-00340-1

**Published:** 2021-11-11

**Authors:** Katarzyna Z. Kuter, Maria Śmiałowska, Krystyna Ossowska

**Affiliations:** 1 Department of Neuropsychopharmacology, Maj Institute of Pharmacology, Polish Academy of Sciences, 12 Smetna St., 31-343 Kraków, Poland; 2 Department of Neurobiology, Maj Institute of Pharmacology, Polish Academy of Sciences, Kraków, Poland

**Keywords:** Parkinson’s disease, Paraquat, Pesticides, Oxidative stress, Inflammation, Microglia, α-Synuclein, Synphilin-1, Neurodegeneration, Substantia nigra, Ventral tegmental area, Midbrain

## Abstract

**Background:**

Prolonged inflammation, oxidative stress, and protein aggregation are important factors contributing to Parkinson’s disease (PD) pathology. A known ROS generator, pesticide paraquat (PQ), was indicated as an environmental substance potentially increasing the incidence of PD and is used to model this disease. We investigated if a combination of inflammation and oxidative stress in subthreshold doses would exacerbate the modelled neuropathology.

**Methods:**

We examined the late effects of acute or repeated peripheral inflammation induced by low dose of LPS (10 μg/kg, *ip*) on PQ toxicity in the rat nigrostriatal dopaminergic pathway, microglial activation markers and expression of major Lewy bodies proteins, α-synuclein and synphilin-1.

**Results:**

We observed that LPS increased, while PQ decreased body temperature and microglia CD11b expression in the SN. Single LPS pretreatment, 3 h before repeated weekly PQ injections (4×) slightly aggravated neuronal degeneration in the SN. Moreover, degeneration of dopaminergic neurons after weekly repeated inflammation itself (4×) was observed. Interestingly, repeated LPS administration combined with each PQ dose counteracted such effect. The expression of α-synuclein decreased after repeated LPS injections, while only combined, repeated LPS and PQ treatment lowered the levels of synphilin-1. Therefore, α-synuclein and synphilin-1 expression change was influenced by different mechanisms. Concomitantly, decreased levels of the two proteins correlated with decreased degeneration of dopaminergic neurons and with a normalized microglia activation marker.

**Conclusions:**

Our results indicate that both oxidative insult triggered by PQ and inflammation caused by peripheral LPS injection can individually induce neurotoxicity. Those factors act through different mechanisms that are not additive and not selective towards dopaminergic neurons, probably implying microglia. Repeated, but small insults from oxidative stress and inflammation when administered in significant time intervals can counteract each other and even act protective as a preconditioning effect. The timing of such repetitive insults is also of essence.

**Supplementary Information:**

The online version contains supplementary material available at 10.1007/s43440-021-00340-1.

## Introduction

Pesticide paraquat (1,1ʹ-dimethyl-4,4ʹ-bipyridinium dichloride, PQ) is used as a model substance to induce oxidative and energetic stress and progressive degeneration of dopaminergic neurons in animals [1–6]. Long-term use of this compound in agriculture increases the incidence of Parkinson’s disease (PD) in humans [7–11]. PD is a slowly progressing neurodegenerative disorder, which mainly affects dopaminergic neurons in the substantia nigra (SN) of the central nervous system (CNS) [12]. According to the ‘multiple hit’ hypothesis, different factors contribute to neuronal degeneration in idiopathic PD [13, 14]. Among them, aging processes, oxidative stress, energy deficit through mitochondrial inhibition, prolonged inflammation, protein misfolding, and aggregation, are all pointed out as the main pathomechanisms. In post mortem PD patient brains elevated TNF-α, COX-2 and pro-inflammatory IL-1β were found [15, 16], suggesting an ongoing chronic brain inflammatory process. Furthermore, neuroinflammation itself can cause PD symptoms. Head trauma, sepsis, encephalitis, and inflammatory processes in the perinatal period can often result in parkinsonism in old age [15, 17]. It was also proven that even minor, peripheral but long-term inflammation caused by diseases such as, for example, ulcerative colitis, can increase the risk of SN neuron degeneration [18–20]. Among brain structures, SN is known to be rich in microglia and poor in potentially supportive astrocytes, making it particularly susceptible to inflammation [19, 21]. Furthermore, regular use of non-steroid anti-inflammatory drugs is correlated with a lower incidence of PD [22]. All the above observations show that inflammatory processes are important in the pathogenesis of PD. However, it is still uncertain whether inflammation is a primary disease mechanism or is triggered by signals from already diseased neurons. These facts led us to investigate whether inflammatory processes combined with oxidative stress would affect the vulnerability of dopaminergic neurons in the SN.

Furthermore, mutations or multiplication of α-synuclein gene were implicated in familial forms of PD [23]. Proteins such as α-synuclein and synphilin-1 are constituents of Lewy bodies and markers of parkinsonian pathology. α-Synuclein has the ability of self-aggregation to potentially cytotoxic forms. Synphilin-1 interacts with α-synuclein and promotes the formation of cytosolic inclusions [23, 24]. The previous studies indicated that aggregation of α-synuclein was enhanced after oxidative stress [25] and such aggregated forms activated microglia [26]. However, mechanisms of those protein’s aggregation and toxicity have not yet been fully explained.

The combination of oxidative stress induced by exogenous substances and inflammatory processes could additively influence accumulation of α-synuclein and synphilin-1 in Lewy bodies in PD brains. Therefore, in this study, we examined the late effects of acute or repeated inflammation induced by small doses of peripherally injected lipopolysaccharide (LPS) on the toxicity of pesticide PQ in the dopaminergic system of the rat brain.

This study proposes a new approach based on the administration of small doses of LPS and/or PQ. Such treatment alone does not cause general acute strong defects, similarly to slowly progressing PD. The aim was to look closer at the long-lasting effects of LPS and PQ, which more closely resemble an environmental situation in people exposed to pesticides, having minor peripheral inflammation, who slowly develop disease signs over many years.

## Materials and methods

### Animals

Male Wistar rats (Charles Rivers, Germany), 3 months old at the beginning of the experiment, were kept in an artificial light 12 h dark/light cycle (light from 07:00 to 19:00), 4–5 per cage, with free access to food and water. The experiments were carried out in compliance with the Animal Experiments Bill of January 21, 2005; (published in Journal of Laws no. 33/2005, item 289, Poland) and according to the NIH Guide for the Care and Use of Laboratory Animals. They also received approval from the Local Ethics Committee (563/2008). In total, 63 rats were used. All efforts were made to minimize the number of animals and their suffering.

### Drugs and treatment

**Fig. 1:**
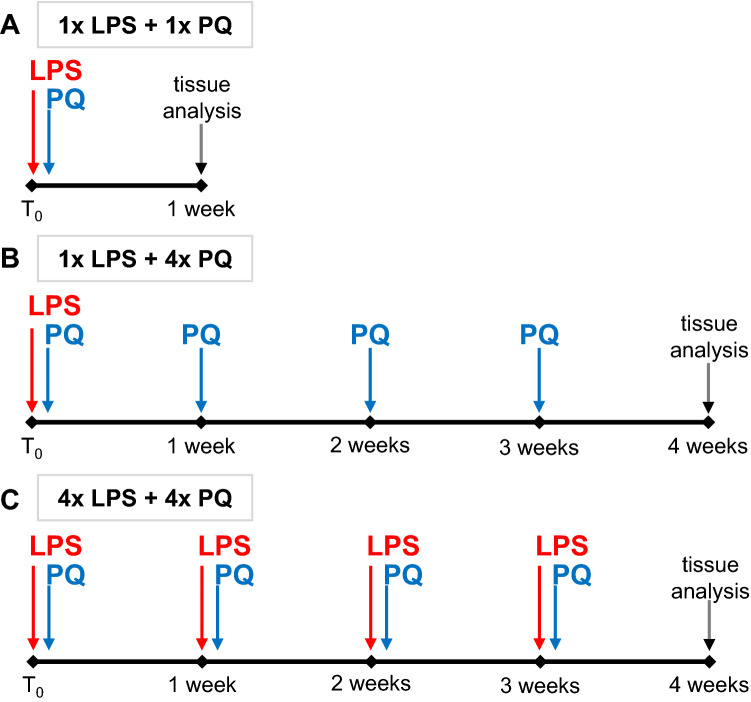
Graphical representation of rat study timelines. Analyses were performed **A** after a single LPS administration (10 µg/kg, ip), 3 h later followed by PQ (10 mg/kg, ip); **B** after a single LPS injection, 3 h later followed by 4 doses of PQ administered once a week; **C** after repeated LPS injection, always 3 h before weekly PQ injections

Paraquat dichloride (PQ, 1,1ʹ-dimethyl-4,4ʹ-bipyridinium dichloride; Sigma-Aldrich, Germany) was dissolved in sterile water and administered *ip* at a dose of 10 mg/kg. LPS (Escherichia coli lipopolysaccharide, serotype 055:B5, Sigma-Aldrich, Germany) was administered at a small dose of 10 μg/kg *ip*, always 3 h before PQ (Fig. 1).

Three sets of experiments were performed (Fig. 1): (i) a single injection of LPS followed by a single injection of injection of PQ; (ii) a single LPS injection followed by 4 doses of PQ administered every 7th day; (iii) 4 LPS injections followed by 4 doses of PQ administered every 7 days. Respective controls received LPS, PQ, or solvent, alone, at the same time. All animals were killed by decapitation 7 days after the last dose of PQ or solvent administered.

### Body temperature measurements

Body temperature was measured inside the rat ear using an infrared light digital thermometer (Geratherm Medical AG, Germany). The air temperature in the exam room was monitored. Each animal was tested immediately before LPS injection—at the beginning of the experiment, to measure the basal body temperature (T_0_). The mean value of three separate measures was used. Next, the measurement was performed 3 h after injection of LPS—immediately before administration of PQ. The last measurement was made after 3 more hours, which was 3 h after PQ administration and 6 h after LPS. Such measurements were made each week during treatment. For statistical analysis, delta T_0_–T_i_ was used.

### HPLC determination of catecholamines in brain homogenates

After decapitation, the left striata were immediately dissected and frozen on dry ice. The tissue was kept at − 80 °C until further analysis. Dopamine (DA) levels and its metabolites: 3,4-dihydroxyphenylacetic acid (DOPAC), 3-methoxytyramine (3-MT), homovanillic acid (HVA) as well as noradrenaline (NA), serotonin (5-HT) together with its metabolite 5-hydroxyindoleacetic acid (5-HIAA) were assessed using the HPLC method with electrochemical detection [27]. Tissue samples were homogenized in 0.1 M perchloric acid containing 0.05 mM ascorbic acid, then centrifuged (10,000×*g*, 15 min, 4 °C), filtered through 0.2 μm cellulose membrane (Alltech Centrifuge Filters, USA), centrifuged again (4,000×*g*, 3 min, 4 °C) and injected into the system that consisted of thermally controlled ASI-100 autosampler (4 °C), P680 isocratic pump with degasser (Dionex, Germany), column (Hypersil Gold C18, 150 × 3.0 mm, 3 μm, Thermo Scientific, UK), TCC-100 thermal controlled column compartment (32 °C, Dionex, Germany) and electrochemical detector analytic cell 5010 Coulochem III (ESA, Inc. USA). The mobile phase consisted of 50 mM NaH_2_PO_4_ × 2H_2_O; 40 mM citric acid; 0.25 mM 1-octanesulfonic acid sodium salt; 0.25 mM EDTA; 1.3% acetonitrile; 2.4% methanol. The flow rate was 0.8 ml/min. The applied potential was E1 = − 175 mV and E2 =  + 350 mV. Data were quantified using the area under the peak and external standards with Chromeleon software (Dionex, Germany). They are presented as ng/g of wet tissue. The turnover rates of amines were calculated as metabolite-to-neurotransmitter ratios.

### Immunohistochemistry and histology

The caudal parts of the brains were rapidly removed, post-fixed in cold 4% paraformaldehyde for 7 days, and cryoprotected in a 20% sucrose solution in phosphate buffered saline (PBS) until they sank. The brains were then cut on a freezing microtome into 30 μm frontal sections according to stereological rules. Each 6^th^ section was sampled into a series of sections that covered all lengths of the SN and adjacent series were stained as previously described [28]. Free-floating sections were incubated for 48 h at 4 °C in a primary antibody (anti-tyrosine hydroxylase (TH), 1: 3,000; anti-α-synuclein, 1:4,000; anti-synphilin-1, 1:3,000; (all from Chemicon Int.; Millipore, USA) and anti—CD11b, 1:500 AbD Serotec, UK, anti-Iba1, 1:2,000, Wako, Japan), rinsed in PBS, then incubated for 30 min in secondary antibodies (anti mouse or anti rabbit biotinylated, 1:200, Vector Laboratories, UK) and processed using an ABC-peroxidase kit (Vector Laboratories, UK) and 3,3ʹ-diaminobenzidine as a chromogen. The stained sections were mounted on slides, dried, stained with 1% cresyl violet (CV) and covered. TH immunoreactive (TH + /CV +) dopaminergic neurons and non-DA neurons (TH −/CV +) were counted stereologically in the SN and ventral tegmental area (VTA).

### Stereology

The stained cells were counted stereologically in the bilateral SN pars compacta (SNc) as previously described [2]. The tissue sampling was random, starting from the beginning of the structure, systematic and uniform, and every sixth section was taken from all SNc. At least 8–10 sections were sampled throughout the length of the structure.

Stereological counting was performed using a light microscope (Leica, Denmark) equipped with a projecting camera (Basler Vision Technologies, Germany) and an *xyz* stage stepper (PRIOR ProScan) controlled by newCAST software (Visiopharm, Denmark). The analyzed regions were outlined under lower magnification (5x), and their areas were estimated. The number of stained cells was calculated at 63 × magnification using a randomized meander sampling and optical dissector method. The dissector height was 10 μm. The area of the counting frame was 8302.8 μm^2^ and covered 30% of the screen area. Meander sampling probed 10% (for TH + /CV +) or 7% (for TH −/CV +) or 5% (for Iba1 +) of the delineated regions of interest, resulting in counting not less than 150 cells from each animal. On double stained (TH/CV) sections non-dopaminergic neurons were identified with large cell bodies stained only with CV, not TH. Astrocytes visible as small CV + nuclei were not counted. Microglial Iba1 + cell bodies were counted. Coefficiency error was smaller than half of the observed coefficiency of variation [29].

### Densitometric analysis of immunostaining

The sections stained immunohistochemically for α-synuclein, synphilin-1, CD11b and Iba1 were analyzed densitometrically in the region of SN (compacta and reticulata) outlined under camera magnification. Relative density units (ROD) were counted using an image analysis system (MCID, St. Catharines, Ontario, Canada). The background signal was subtracted from each section separately, close to the region of interest. The results were presented as the mean of each ROD value minus background.

### Statistical analysis

Results are presented as means ± standard errors of mean (SEM). Statistical analysis of the results was performed using STATISTICA 8.0 software (StatSoft Inc., USA). *p* ≤ 0.05 was considered as statistically significant and 0.1 ≥ *p* ≥ 0.05 were considered as trends.

The data were analyzed using two-way factorial ANOVA with Fisher’s Least Significant Difference post hoc test, except for body temperature results, where a repeated measures ANOVA was performed. For experiments with 1 and 4 doses of LPS, the same control groups (SOLV + SOLV and SOLV + 4×PQ) were used and are presented on the same graph. All groups consisted of 6–8 animals.

## Results

### The effect of single or repeated LPS and PQ administration on body temperature

All animals were first injected with LPS (or solvent) and 3 h later with PQ (or solvent) (Fig. 1). Body temperature was measured before each of these treatments and after a further 3 h.

**Fig. 2:**
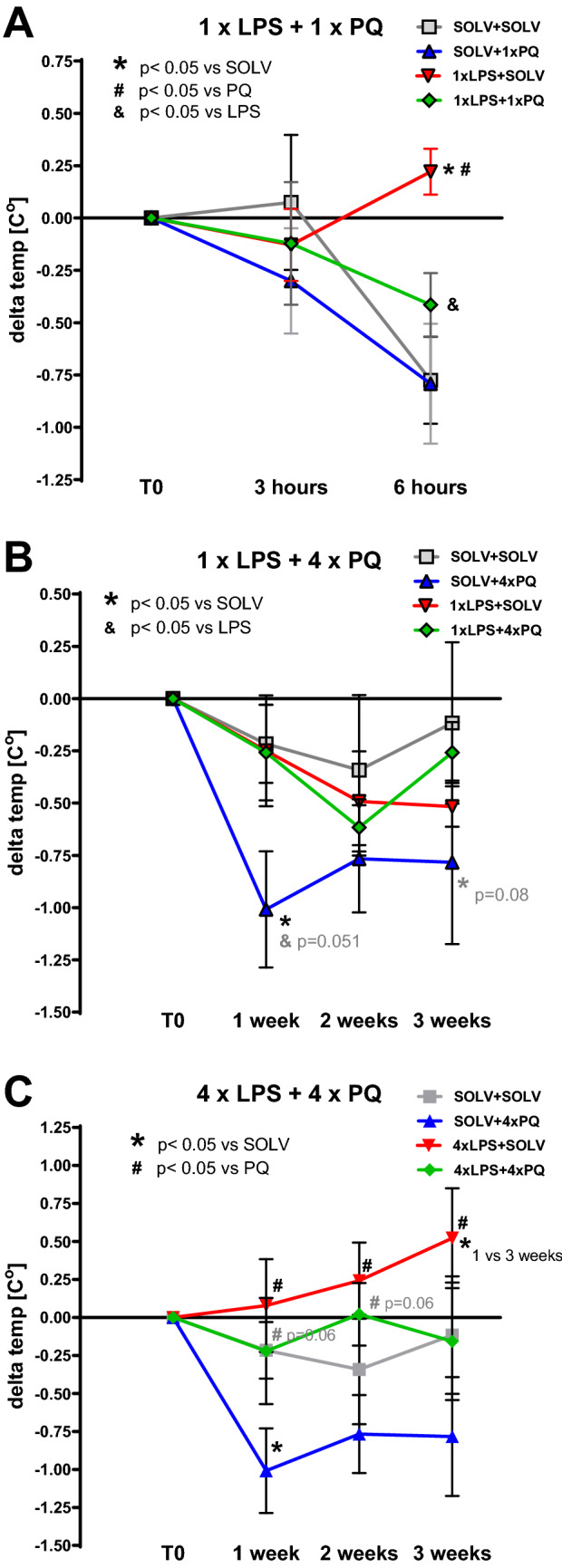
Changes in the Wistar rat body temperature **A** measured after the first injection of LPS and/or PQ. First measurement—immediately before LPS (or solvent) injection (T0), second—3 h later, immediately before PQ (or solvent) injection, third—3 h later. Changes in the body temperature measured after single (**B**) or repeated (**C**) injections of LPS and/or PQ, always 7 days after treatment. The values show mean from 3 separate measures and delta between T0 and subsequent measure. Two-way repeated measures ANOVA with Fisher Least Significant Difference post hoc test, *p* ≤ 0.05 vs solvent (*),vs PQ (#), vs LPS (&). Trends with 0.05 ≤ *p* ≤ 0.1 are marked in grey. 1×SOLV + 1×SOLV *n* = 8; 1×SOLV + 1×PQ *n* = 5; 1×LPS + 1×SOLV *n* = 6; 1×LPS + 1×PQ *n* = 6; 4×SOLV + 4×SOLV *n* = 6; 4×SOLV + 4×PQ *n* = 6; 1×LPS + 4×SOLV *n* = 6; 1×LPS + 4×PQ *n* = 6; 4×LPS + 4×SOLV *n* = 7; 4×LPS + 4×PQ *n* = 7

The single injection of a small dose of LPS alone (10 μg/kg, *ip*) (F_3,22_ = 4.09, *p* = 0.019) increased the body temperature of the rat measured after 6 h (SOLV + SOLV *t* = 35.65 ± 0.23 °C vs 1×LPS + SOLV *t* = 36.26 ± 0.11 °C; *p* = 0.032) (Fig. 2A). Single treatment with PQ (10 mg/kg *ip*) non-significantly lowered body temperature, but combined treatment with 1 dose of LPS + PQ significantly decreased the response to immunostimulant (1×LPS + SOLV, *t* = 36.26 ± 0.11 °C vs 1×LPS + 1×PQ *t* = 36.06 ± 0.17 °C; *p* = 0.048).

On the other hand, single LPS alone treatment did not induce any long-lasting effect after 7 days of withdrawal (Fig. 2B), but repeated weekly 4 doses of LPS (F_3,22_ = 2.66, *p* = 0.07) tended to increase body temperature over time (*p* = 0.056 for 1 week vs 3 weeks) (Fig. 2C). Repeated injections with PQ alone decreased body temperature when measured 7 days after treatment (*p* = 0.04 after 1 week and *p* = 0.08 after 3 weeks) compared to the SOLV control (Fig. 2B). When PQ treatment was preceded by a single LPS injection, the effect was diminished. Changes induced by 4×PQ alone and 4×LPS alone were statistically significantly different – LPS increased body temperature and PQ decreased it in the long term. Combined, repeated LPS and PQ treatments counteracted the changes observed with LPS or PQ alone, normalizing body temperature.

### The effect of single or repeated LPS and PQ treatment on microglia activation

**Fig. 3:**
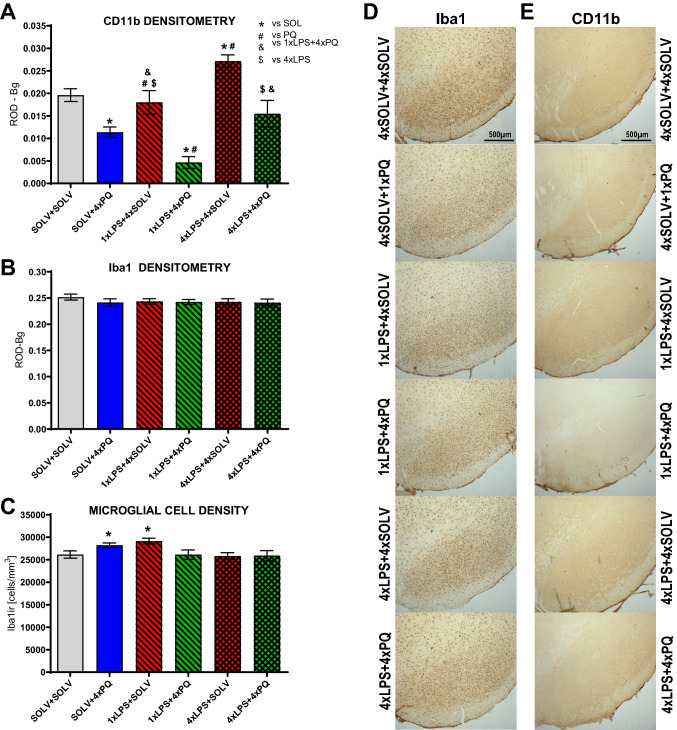
The expression of microglia markers. Densitometric analysis of CD11b—marker of activated microglia (**A**), Iba1—constitutive microglia marker (**B**) and stereological counting of Iba1 + cells (**C**) in the substantia nigra (SN) of Wistar rats treated with single or repeated LPS followed by four weekly PQ injections. Results are shown as mean relative optical densities with subtracted background levels (A and B) and as mean cell number per mm^3^ (**C**). Two way ANOVA with Fisher Least Significant Difference post hoc test, *p* ≤ 0.05 marked as significant vs solvent (*), vs PQ (#), vs 4×LPS ($), vs 1×LPS + 4×PQ (&). Representative staining of Iba1 (**D**) and CD11b (**E**) form each group. SOLV + SOLV *n* = 6/6/8; SOLV + 4×PQ *n* = 6/6/6; 1×LPS + 4×SOLV *n* = 6/6/6; 1×LPS + 4×PQ *n* = 6/6/6; 4×LPS + 4×SOLV *n* = 5/7/7; 4×LPS + 4×PQ *n* = 6/7/7, for A, B, C, respectively

To check the ability of low-dose LPS intraperitoneal administration to induce brain microglia activation, we analyzed the intensity of immunostaining for CD11b in the SN (Fig. 3A) (F_5,29_ = 14.27, *p* ≤ 0.001). The single administration of LPS followed by four weekly doses of solvent did not change staining. Interestingly, repeated PQ injections decreased the expression of the activated microglia marker. This effect was intensified when four doses of PQ were preceded by a single LPS dose. On the other hand, four repeated injections of LPS alone increased the level of the microglia marker CD11b. Four doses of combined LPS and PQ treatment decreased the effects of LPS or PQ alone, normalizing the microglia state. The observed effects were counteracted by each other, similarly as in body temperature measurements. The effect of single vs repeated LPS administration in combination with PQ was significantly different.

To check whether the observed changes were not caused by microglia death or a change in their number, we also analyzed the optical density of immunostaining for the constitutive microglial marker Iba1 (Fig. 3B) and stereologically counted Iba-1 + microglia cells (Fig. 3C). Both did not show deficits.

### The effect of LPS and PQ administration on neurodegeneration in the SNc and VTA

**Fig. 4:**
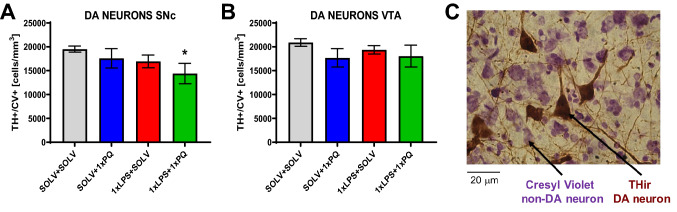
The stereological counting of tyrosine hydroxylase (TH) immunoreactive neurons in the substantia nigra pars compacta (SNc) (**A**) or ventral tegmental area (VTA) (B) after single dose of LPS and/or PQ and representative staining of TH + and CV + (cresyl violet) neurons (**C**) from control Wistar rat. Results are shown as mean cell number per mm^3^. Two way ANOVA with Fisher least significant difference post hoc test, *p* ≤ 0.05 marked as significant vs solvent. SOLV + SOLV *n* = 8; SOLV + 1×PQ *n* = 5; 1×LPS + 1×SOLV *n* = 6; 1×LPS + 1×PQ *n* = 6

Neither a single dose of PQ nor LPS alone did not reduce the number of dopaminergic cells in the SNc or VTA when analyzed 7 days later. However, already a single coadministration of PQ and LPS induced a 26% decrease in the density of TH + /CV + neurons detected in the SNc one week after treatment (SOLV + SOLV 19,514 ± 637 vs 1×LPS + 1×PQ 14,398 ± 2131; *p* = 0.014) (Fig. 4A). Despite slight reductions, no significant changes were observed in the VTA (Fig. 4B).

**Fig. 5:**
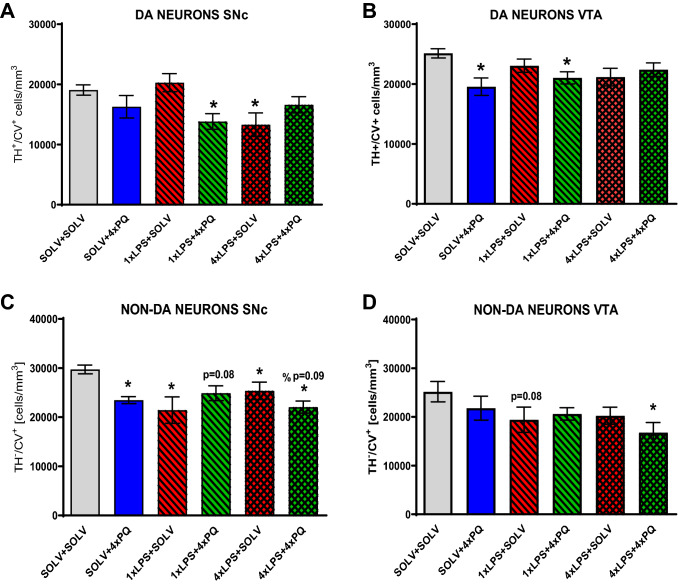
The stereological counting of dopaminergic (TH + /CV +) (**A**, **B**) and non-dopaminergic, (TH-/CV +) (**C**, **D**) cells in the substantia nigra pars compacta (SNc) (**A**, **C**) and ventral tegmental area (VTA) (**B**, **D**) after single or repeated LPS treatment followed by four weekly PQ injections in Wistar rat. Results are shown as mean cell number per mm^3^. Two way ANOVA with Fisher Least Significant Difference post hoc test, *p* ≤ 0.05 marked as significant vs solvent (*), vs LPS (%). 0.1 ≥ *p* ≥ 0.05 were considered as trends. SOLV + SOLV *n* = 4/4/5/5; SOLV + 4×PQ *n* = 4/5/4/5; 1×LPS + 4×SOLV *n* = 5/6/6/6; 1×LPS + 4×PQ *n* = 5/5/5/6; 4×LPS + 4×SOLV *n* = 6/7/7/7; 4×LPS + 4×PQ *n* = 7/7/6/7, for A, B, C, D, respectively

Four weekly doses of PQ did not reduce the number of TH + /CV + neurons statistically but reduced the number of non-dopaminergic neurons (TH −/CV +) in the SNc after 4 weeks of treatment, showing nonspecific toxicity of PQ (Fig. 5A, C) (F_3,18_ = 10.98, *p* = 0.003). The opposite situation was observed in the VTA, the density of dopaminergic neurons was reduced (F_3,16_ = 3.99, *p* = 0.027) but no changes were observed in the non-dopaminergic neurons (Fig. 5B, D).

A single injection of LPS caused a decrease in non-dopaminergic neuron density in the SNc (F_3,16_ = 6.46, *p* = 0.02) and a tendency to decrease non-dopaminergic neurons in the VTA (*p* = 0.08) (F3,18 = 1.12, *p* = 0.30) (Fig. 5CD). This effect was visible only 4 weeks after treatment.

Single pretreatment with LPS, followed by four doses of PQ also decreased the density of TH + /CV + neurons in the SNc (by 27.5%, SOLV + SOLV 19,070 ± 858 vs 1×LPS + 4×PQ 13,835 ± 1305; *p* = 0.003) and VTA. Non-dopaminergic neurons showed a decreasing trend (*p* = 0.08) in the SNc.

After four doses of LPS alone, a decrease was observed in TH + /CV + (SOLV + SOLV 19,070 ± 858 vs 4×LPS + SOLV 13,300 ± 1960; *p* = 0.025) as well as in TH-/CV + neurons in the SNc. VTA was not affected. Interestingly, after combined administration of 4 × LPS and PQ, a significant loss was documented only in non-dopaminergic neurons in both SNc and VTA.

Neither oxidative stress caused by PQ nor inflammation induced by LPS were selective processes for dopaminergic neuron degeneration.

### The influence of acute and repeated LPS and PQ administration on the levels of neurotransmitters and their metabolism in the striatum

One week after a single exposure of rats to LPS and/or PQ, only small changes in DA metabolism were observed (increased DOPAC (F_3,21_ = 9.66, *p* = 0.005) and DOPAC/DA ratio (F_3,21_ = 12.36, *p* = 0.002)). Similarly, serotoninergic metabolism was increased in response to LPS or to PQ (Table 1).

**Table 1:** HPLC analysis of catecholamines after acute treatment with LPS and/or PQ

	SOLV + SOLV		SOLV + 1×PQ		1×LPS + SOLV		1×LPS + 1×PQ	
	Mean	SEM	Mean	SEM	Mean	SEM	Mean	SEM
*n*	8		5		6		6	
DA	10,389.08	304.67	9443.06	716.92	9802.28	365.41	10,465.33	405.24
DOPAC	983.38	42.12	930.53	61.28	**1097.93#**	**46.45**	1123.13*	**49.09**
HVA	925.30	52.16	953.24	48.43	928.51	84.54	1067.80	58.02
3-MT	314.62	38.31	255.37	31.16	286.77	44.68	262.77	30.62
DOPAC/DA	0.0946	0.0027	0.0997	0.0062	0.1120*	**0.0021**	0.1075*	**0.0032**
HVA/DA	0.0892	0.0050	0.1028	0.0075	0.0943	0.0065	0.1025	0.0056
3-MT/DA	0.0309	0.0046	0.0272	0.0028	0.0289	0.0042	0.0248	0.0022
5-HT	389.53	20.80	401.33	24.33	418.25	20.25	398.72	17.53
5-HIAA	363.65	19.46	**407.27#**	**16.26**	439.38*	**13.09**	449.02*	**14.65**
5-HIAA/5-HT	0.9484	0.0591	1.0197	0.0220	1.0674	0.0781	1.13*	**0.0357**
NA	47.23	4.32	46.97	6.15	56.37	6.03	46.42	4.78

HPLC analysis of dopamine (DA), its metabolites 3,4-dihydroxyphenylacetic acid (DOPAC), 3-methoxytyramine (3-MT), homovanillic acid (HVA) and turnover rates (DOPAC/DA, HVA/DA and 3-MT/DA) as well as serotonin (5-HT), its metabolite 5-hydroxyindoleacetic acid (5-HIAA), turnover rate (5-HIAA/5-HT) and noradrenaline (NA) in the striatum of rats treated with single LPS followed by single PQ injection. Results are shown as mean in ng/g of wet tissue. Two way ANOVA with Fisher Least Significant Difference post hoc test, *p* ≤ 0.05 marked as significant vs solvent (*) and trends with *p* = 0.09 vs solvent (#)

**Fig. 6:**
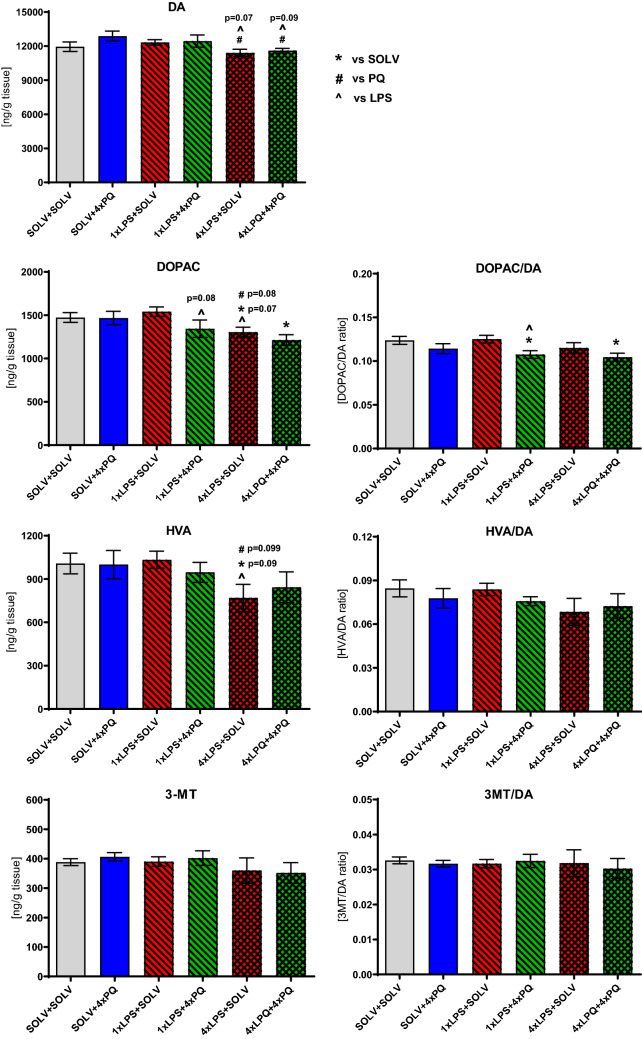
HPLC analysis of dopamine (DA), its metabolites 3,4-dihydroxyphenylacetic acid (DOPAC), 3-methoxytyramine (3-MT), homovanillic acid (HVA) and turnover rates (DOPAC/DA, HVA/DA and 3-MT/DA) in the striatum of rats treated with single or repeated LPS followed by four weekly PQ injections in Wistar rat. Results are shown as mean in ng/g of wet tissue. Two way ANOVA with Fisher least significant difference post hoc test, *p* ≤ 0.05 marked as significant vs solvent (*), vs PQ (#),vs LPS (^). 0.1 ≥ *p* ≥ 0.05 were considered as trends. All groups consisted of 6–7 animals. Exact numbers of samples in each group are indicated in Table 2

Four doses of PQ given 7 days apart decreased only the 5-HT turnover rate (5-HIAA/5-HT SOLV + SOLV 0.9252 ± 0.0184 ng/g vs SOLV + 4×PQ 0.8500 ± 0.0214 ng/g; *p* ≤ 0.05) (Fig. 6). Single treatment with LPS alone, 4 weeks prior to decapitation, also decreased only 5-HIAA/5-HT rate (SOLV + SOLV 0.9252 ± 0.0184 ng/g vs 4×LPS + SOLV 0.8548 ± 0.0282 ng/g; *p* ≤ 0.05). Rats pretreated with LPS before 4 doses of PQ tended to decrease DOPAC levels (*p* = 0.08) and decreased the DOPAC/DA ratio, as well as 5-HIAA/5-HT in the striatum (*p* ≤ 0.05) (Fig. 6, Table 2). A single exposure to the LPS dose before 4 consecutive doses of PQ showed more effects than either of these factors alone, suggesting aggravation of the toxicity, similar to the count of neurons.

**Table 2:** HPLC analysis of serotonin (5-HT), its metabolite 5-hydroxyindoleacetic acid (5-HIAA), turnover rate (5-HIAA/5-HT) and noradrenaline (NA) levels in the striatum of rats treated with single or repeated LPS followed by four weekly PQ injections

	SOL + SOL		SOL + 4×PQ		1×LPS + SOL		1×LPS + 4×PQ		4×LPS + SOL		4×LPS + 4×PQ	
	Mean	SEM	Mean	SEM	Mean	SEM	Mean	SEM	Mean	SEM	Mean	SEM
*n*	6		6		6		6		7		7	
5-HT	629.7 ± 39.8		648.6 ± 25.6		629.5 ± 29.5		689.6 ± 27.7		535.8 ± 23.15*^#^**^**		510.0 ± 26.04*^#^**^**	
5-HIAA	581.0 ± 33.7		550.4 ± 21.4		516.8 ± 34.1		554.0 ± 20.3		456.2 ± 18.73*^#^		434.0 ± 38.22*^#^**^**	
5-HIAA/5-HT	0.93 ± 0.02		0.85 ± 0.02*		0.82 ± 0.04*		0.81 ± 0.02*		0.85 ± 0.03		0.84 ± 0.04	
NA	51.4 ± 16.7		65.1 ± 24.4		43.6 ± 10.1		51.6 ± 5.8		40.7 ± 16.9		38.6 ± 8.8	

Results are shown as mean in ng/g of wet tissue. Two way ANOVA with Fisher least significant difference post hoc test, *p* ≤ 0.05 marked as significant vs solvent (*), vs PQ (#), vs LPS (^). 0.1 ≥ *p* ≥ 0.05 were considered as trends

Interestingly, single pretreatment with LPS followed by a single injection of PQ increased the DA and 5-HT metabolism measured by the DOPAC/DA and 5-HIAA/5-HT ratio, but single administration of LPS when followed by repeated administration of PQ decreased those parameters (Table 1).

Repeated administration of small doses of LPS (10 μg/kg) alone was intended to induce minor chronic peripheral inflammation. Such treatment resulted in decreased levels of DA metabolites DOPAC (F_3,22_ = 11.08, *p* = 0.003) and HVA (F_3,22_ = 4.33, *p* = 0.049) in the brain (11.5%, *p* = 0.07 and 23.6%, *p* = 0.09) but not turnover rates (Fig. 6). 5-HT levels were also decreased, together with its metabolite 5-HIAA, but again not its turnover rate (Table 2). There was no enhancement of the LPS effect due to co-administration with PQ. Only the DOPAC/DA ratio was decreased, reflecting the effect observed after 4 doses of PQ pretreated with a single dose of LPS. When LPS pretreatment was repeated each time before 4 doses of PQ—no aggravation of the effect was observed. Furthermore, repeated treatment with LPS alone showed stronger influence on neurotransmitter levels and metabolism than 4 doses of PQ itself. The results indicate that repeated LPS and PQ counteracted each other’s effects.

### The influence of LPS and PQ administration on the expression of α-synuclein and synphilin-1 in the SN

**Fig. 7:**
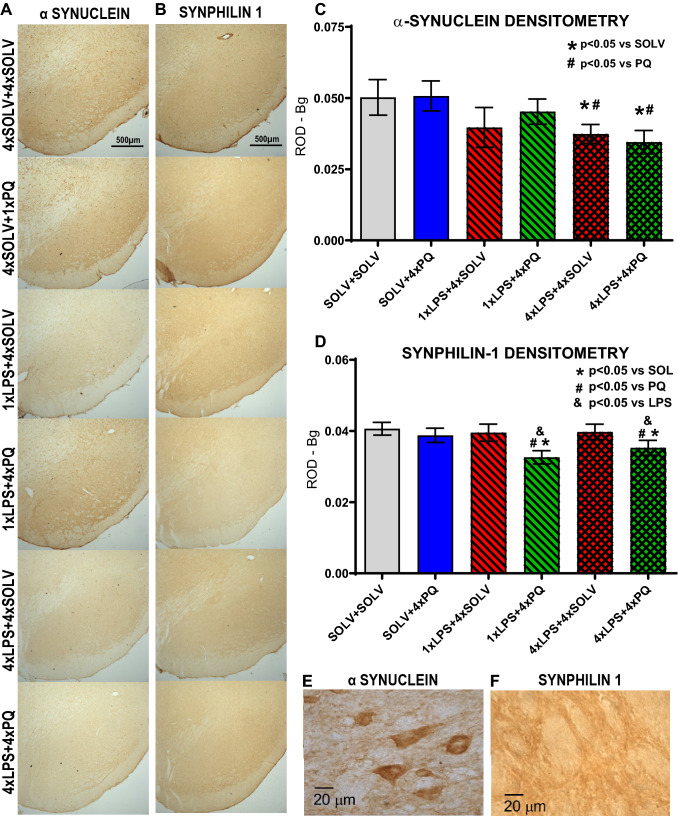
The expression of Lewy bodies markers. Representative staining of substantia nigra (SN) for α-synuclein (**A**, **E**) and synphilin-1 (**B**, **F**). Densitometric analysis of α-synuclein (**C**) and synphilin-1 (**D**) in the substantia nigra of Wistar rats treated with single or repeated LPS followed by four weekly PQ injections. Results are shown as mean relative optical densities with subtracted background levels. Two way ANOVA with Fisher Least Significant Difference post hoc test, *p* ≤ 0.05 marked as significant vs solvent (*), vs PQ (#), vs LPS (&). SOLV + SOLV *n* = 6; SOLV + 4×PQ *n* = 6; 1×LPS + 4×SOLV *n* = 6; 1×LPS + 4×PQ *n* = 6; 4×LPS + 4×SOLV *n* = 6 and 8; 4×LPS + 4×PQ *n* = 6 and 8, respectively for** C** and** D**

The immunostaining for α-synuclein was widespread in the brain structures and visible both inside cells, probably neurons, as suggested by the shape and size of the cells, as well as in the neuropil. The intensity of α-synuclein expression was measured within the entire SN (both *pars* *compacta* and *reticulata*). Only repeated administration of LPS reduced the intensity of immunostaining for α-synuclein in the SN (F_3,22_ = 11.5, *p* = 0.002) (Fig. 7A). The effect was observed both after 4 doses of LPS alone and after combined treatment with 4 doses of PQ. The single administration of LPS also tended to decrease the expression of α-synuclein but it was not statistically significant.

Furthermore, only combined treatment with LPS and PQ decreased the intensity of immunostaining for synphilin-1—both after single and repeated LPS (F_3,20_ = 3.244, *p* = 0.08) (Fig. 7B). Neither LPS nor PQ alone did not change the expression of this protein.

## Discussion

### LPS and PQ induce opposite inflammatory reactions

This study was planned to check if minor inflammatory processes and oxidative insults, that often pass unattended with medical help, could contribute to the slowly progressing neurodegenerative processes, similar to PD. Therefore, the doses of LPS (10 μg/kg) used here were small, as compared to the previous studies (1–7 mg/kg, own data not shown and [30]), and did not induce any sickness behavior.

The body temperature changes dynamically in time during inflammation, hence in the present study the temperature was measured across time. Whereas LPS injection increased body temperature, the treatment with PQ resulted in consistent decrease in body temperature (see Fig. 2). Such opposite effects resulted in normalized body temperature after combined LPS and PQ treatment, both after single and repeated LPS pretreatment. One could instantly think of hypothermia which was previously shown to be neuroprotective in many neurodegenerative disease models [31–33]. In our study the observed differences in body temperature were rather small but long-lasting (delta T approximately by 1 °C). Previous studies in mice reported only transient hypothermia after PQ (2 × 10 mg/kg/week for 3 weeks) [34]. Interestingly, the parkinsonism-inducing neurotoxin 1-methyl-4-phenyl-1,2,3,6-tetrahydropyridine (MPTP) given *ip* to mice also induced long-lasting hypothermia [35, 36]. Furthermore, PQ generates ROS in a cyclic reaction using NADPH and in this way exhausts energy metabolism reserves. Such mechanism could directly affect body temperature and partially explain the impact of PQ on immune cells mobilization.

### Microglia activation

In the present study, we observed that the changes in rat’s body temperature corresponded with microglia activation markers in the SN. Decreased body temperature after 4 doses of PQ alone lowered expression of microglia activation markers, while repeated LPS caused temperature increase and increased CD11b expression. Interestingly, repeated concominant treatment with LPS and PQ showed normalized microglia CD11b level. This proves that four, repeated concominant injections of LPS and PQ counteracted the effects evoked by inflammatory processes.

Previous studies on microglial cultures showed no changes in microglia activation markers after PQ [37, 38]. On the contrary, in mice injection of PQ (10 mg/kg,* ip*) increased CD11b expression, showing activation of microglia, but those animals were killed earlier than in our study—2 or 4 days after the last dose [39, 40]. Interestingly, more frequent dosing of PQ (10 mg/kg, every 3rd day) was also shown to increase activation of CD11b + microglia in mice [41]. Other studies reported activation of microglia after PQ, already after a single injection [42], but it was tested in combination with maneb [43]. Cicchetti et al. identified some activation of microglia in the SNc 24 h after the last of 8 doses of PQ administered in rats [6]. The question still remains if observed microglia activation was due to the pesticide or to the PQ-induced neurodegeneration. Importantly, all the above experiments applied more intense regimens than in our study and analyzed tissue after shorter times. Therefore, the time schedule of injections plays an important role in inflammatory response to pesticides. Our experiment, performed on Wistar rats employed prolonged, low-level exposure treatment with four doses of PQ given *ip*, 7 days apart, and showed an opposite long-term effect. Interestingly, in our practice, C57BL/6 mice after the dose of PQ (10 mg/kg ip) used also by the other authors [39, 40, 42], showed severe sickness behavior and most of them soon died (own unpublished data). Rats, on the other hand, displayed no such symptoms with the same dose, suggesting that PQ is much more toxic for mice than rats. It would explain some of the discrepancies in microglia activation by PQ between studies in different species. Our data does not contradict the previous results analyzing shorter times post treatment. We studied the long-term changes, since the literature suggested that the prolonged microglia activation might be the reason of dopaminergic cell degeneration in PD [44, 45]. It is rather not the case in relatively short-term administered PQ toxicity.

CD11b is expressed not only in microglia but also in myeloid-lineage cells such as monocytes/macrophages, neutrophils, eosinophils, and basophils or NK cells. LPS pretreatment could also affect BBB permeability. Therefore, one might hypothesize that observed here changes in CD11b expression could be partially also BBB-related. It could increase PQ penetration to the brain and modulate inflammatory cells mobilization but in this study LPS showed rather preconditioning, counteracting effects after repeated doses.

Furthermore, the toxicity of PQ towards microglial cells was reported previously [46, 47]. In our studies despite that PQ always decreased the expression of CD11b protein, it never decreased the number of microglial cells stained for Iba1. Interestingly, microglial cells activated with IFN-gamma were reported to induce neurogenesis, while LPS activated microglia to inhibit it [48]. Thus, depending on the immunological context, differentially activated microglia can influence either survival or degeneration of neurons—what we observed also in our study (see also [49–51]. This issue needs further studies.

### Neurotoxicity of LPS and PQ

In this study, we examined whether induction of acute or repeated peripheral inflammation by a low dose of LPS would affect neuronal survival and influence the neurotoxicity evoked by PQ. Indeed, already a single dose of LPS decreased the density of non-dopaminergic cells in the SNc and VTA when counted after 4 weeks, demonstrating prolonged neurodegeneration due to the small peripheral inflammation itself.

Our results also showed degeneration of dopaminergic neurons in the SNc 4 weeks after treatment with one dose of LPS, preceding PQ injection (1× and 4×) (Fig. 5). Interestingly, no such decrease was observed when each of the 4 PQ injections was administered after LPS. One of the possible explanations for such results could be decreased expression of the TH marker protein rather than actual neuronal cell death. Therefore, when analyzing repeated PQ effects, we counted both TH + and TH − cells, proving the actual loss of neurons. The second explanation of the above discrepancy is the difference in the time of analysis. In the experiment where animals were treated with a single dose of LPS and PQ tissue was collected after 1 week (1×LPS + 1×PQ), while in the experiment with repeated injections tissue was collected after 4 weeks from the first dose (4 × LPS and/or 4 × PQ). Slowly progressing degeneration needs time to evolve, therefore after 4 weeks it could have been easier to detect cell loss. It also seems that the first LPS injection could trigger some dangerous processes, but the subsequent repeated doses might render the system resistant to the following insults, as a preconditioning effect. Longer duration of the experiment allowed for such adaptation. These results could suggest that both LPS and PQ induce a very slow, progressing degeneration process and the timing of oxidative vs inflammatory small insults is critical to trigger neurodegeneration or endogenous protection. This hypothesis should be further tested in the context of idiopathic PD. It can be also suggested that the immunocompetent microglial cells also participate in this differential effect of treatment.

Neither LPS nor PQ induced cell type specific neurodegeneration. Some differences were also observed between SNc and VTA, suggesting that VTA might be more resistant to inflammatory and oxidative stress. This is in line with experimental studies and the lower vulnerability of those neurons in the course of PD, for example, due to the higher expression of the astrocytic growth factor GDF15 [52].

Our results showed that four small doses of LPS, given 7 days apart, not only decreased neuron density, but also tended to reduce DA and its metabolites DOPAC and HVA in the striatum. This indicates that neurodegeneration of dopaminergic neurons can be observed after prolonged inflammation itself. It is in line with previous studies [15–17] after a small peripheral inflammation caused by ulcerative colitis [20, 53, 54]. Possibly, increased BBB permeability due to LPS-induced inflammation could have increased PQ penetration into the brain and aggravated its toxicity. On the other hand, PQ given *ip* passes into the brain in small amounts, but can accumulate after repeated dosing [1, 55–57].

### Preconditioning—effect depending on the time line

The results of our study indicate that single *ip* LPS pretreatment aggravated the toxicity of PQ injected later weekly for 4 weeks. But when LPS was administered prior to each of the 4 PQ injections, we observed improvement in the survival of dopaminergic neuron in the SNc and VTA. Interestingly, other authors have also shown that ongoing inflammation prior to toxic insult, such as induced by PQ, can both prime the degeneration or prevent it, depending on the time between the exposition and the doses used. Mangano and Hayley also reported that exposure of mice to 10 mg/kg PQ (*ip*) 2 days after intranigral injection of LPS pronounced its toxicity, but when administered 7 days after LPS, neuroprotection was observed [58]. Although the intranigral LPS injection is an invasive method, the observed effect was very similar to our results. The small insult induces endogenous protective mechanisms such as the antioxidative response, which is responsible for the preconditioning effect. Our results may be surprising at first, but suggest that administration of low dose of LPS and PQ repeated every 7th day, gives enough time to counteract each other toxicity, and initiate endoprotective mechanisms before the next dose. It seems that such treatment might have acted as a preconditioning and made neurons more resistant to subsequent insults, at least for the time length of this study. Our previous research in the PQ model also showed that at the beginning of weekly treatments, compensatory mechanisms were activated in the nigrostriatal and mesocortical dopaminergic pathways, before major neurodegeneration occurred after 6 months of PQ treatment [59, 60]. Other studies also corroborate the preconditioning effect of a small dose of LPS against neurodegeneration [61–64]. It is well recognized that inflammation is a mechanism with strictly regulated negative feedback. In its early phase, pro-inflammatory cytokines are produced, but after some time, healthy organism also produces anti-inflammatory substances to extinguish the otherwise dangerous process [45]. It is known that activated microglia in the early stage can be neurotoxic, but later in the resolving inflammation phase, active microglia have regenerative function. Therefore, the timing of secondary insults and their toxicity may be related to the phenotype of activated microglia. In our study, LPS administered at 7-day intervals induced the inflammation at first, but, since the dosage was quite small, within 7 days, suppressive processes could be activated and overlapped with the toxic effects of the following doses of PQ and LPS. This is one of the probable explanations for why single LPS administration aggravated PQ toxicity, while repeated treatment spared some neurons. In fact, it has been proven before that low concentrations of LPS induced IL-6 secretion from astrocytes in vitro and supported the survival of dopaminergic neurons, while higher concentrations of LPS killed them [65]. Other numerous molecules can also be responsible for this preconditioning effect, such as granulocyte colony stimulating factor (G-CSF) [65]. Report by Mangano et al. showed the neuroprotective action of G-CSF against PQ toxicity in mice, hence corroborating this theory [66].

### Decreased expression of α-synuclein and synphilin-1 in the SN

α-Synuclein is a protein with natively unfolded structure that can form oligomeric protofibrils, which can further aggregate [67]. It is a major component of Lewy bodies in PD. Some forms of α-synuclein can be toxic for the cells. α-Synuclein is taken up by glial cells and can activate them. In our study, repeated induction of the inflammatory response by small doses of LPS reduced α-synuclein staining (see Fig. 7A). The observed results did not correlate with microglia activation, which was counteracted by PQ. Change in α-synuclein expression was not responsive to PQ treatment, but only to repeated LPS.

We also observed decreased expression of synphilin-1 in the SN but only in animals treated with LPS and PQ together. Synphilin-1 is another cytoplasmic protein involved in PD and is found in the core of Lewy bodies. Previous studies have suggested that synphilin-1 is involved in energy homeostasis, binds and regulates the cellular energy molecule, ATP [68, 69]. Synphilin-1 interacts with α-synuclein and promotes its aggregation and formation of cytosolic inclusions. It specifically inhibits degradation of α-synuclein by the 20S proteasome [23, 24]. Very little information is available about the influence of inflammation on the expression of synphilin-1. Smith et al. reported that A53T α-synuclein-induced neuronal degeneration and astroglia reaction were decreased in the brains of mice with co-overexpression of synphilin-1 and also promoted the formation of aggresome-like structures [70].

Most studies so far have been performed in models overexpressing those two proteins or reported increased levels of α-synuclein and synphilin-1 due to different treatments, but not decreases such as observed here. Interestingly, in the repeated LPS and PQ group, which seemed to be partially protected from degeneration of dopaminergic neurons, both α-synuclein and synphilin-1 densities were decreased. This means that the concomitantly changed expression of α-synuclein and synphilin-1 corresponded with the protective processes. Whether it was causative remains to be explored. Each of those proteins is more susceptible to the other type of stress.

### Conclusions

Our results indicate that both oxidative insults triggered by PQ, as well as inflammation caused by repeated small doses of LPS, can individually induce toxicity. A single small dose of peripheral LPS pretreatment aggravated the toxicity of four PQ doses. However, when LPS was administered before each of the 4 PQ injections, it improved dopaminergic neuron survival in the SN and VTA. It shows that LPS and PQ toxicity acts through different mechanisms and they induced counteracting effects on body temperature and microglia activation. The observed effects were small in magnitude but long-term. The timing of small oxidative and inflammatory repetitive insults play a crucial role in triggering their toxic or protective paradigm. This study shows that small insults can act protectively due to the preconditioning effect.

Small, repetitive insults of different nature should be further studied to search for possible ‘multiple hits’ causes of slowly progressing PD.

## Supplementary Information

Below is the link to the electronic supplementary material.

Supplementary file1 (PPT 65020 KB)

## Abbreviations

BBB: Blood–brain barrier
LPS: Lipopolysaccharide
PD: Parkinson’s disease
PQ: Paraquat
ROS: Reactive oxygen species
SN: Substantia nigra
TH: Tyrosine hydroxylase
VTA: Ventral tegmental area
