# Supplementary material for: The influence of preconditioning with low dose of LPS on paraquat-induced neurotoxicity, microglia activation and expression of α-synuclein and synphilin-1 in the dopaminergic system
Source: Pharmacol Rep. 2021 Nov 11;74(1):67–83. doi: 10.1007/s43440-021-00340-1 (PMC8786770; doi:10.1007/s43440-021-00340-1)
Supplement: Supplementary file 1 — Supplementary file1 (PPT 65020 KB) [file 43440_2021_340_MOESM1_ESM.ppt]

## Slide 1
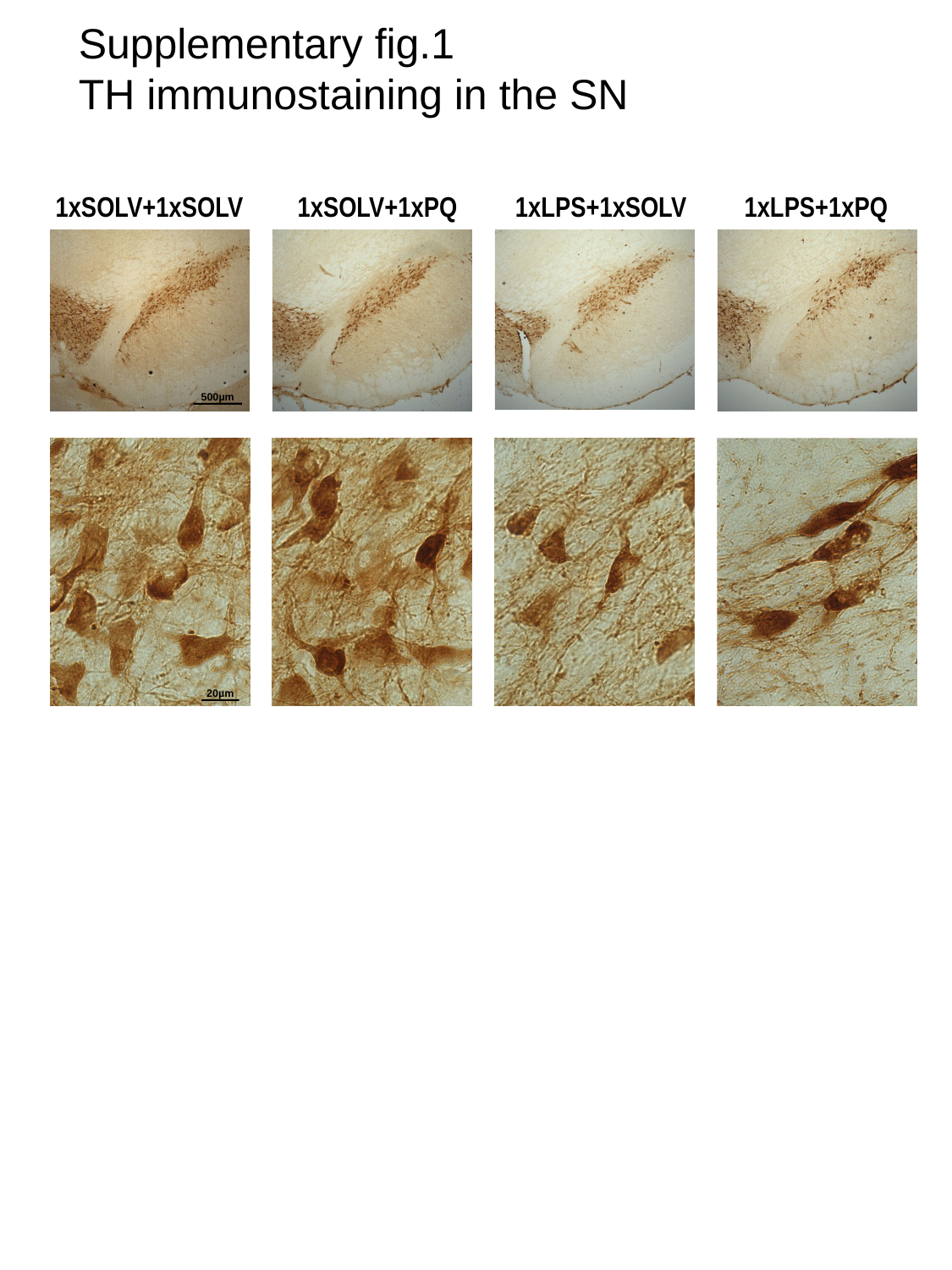

Supplementary fig.1
TH immunostaining in the SN
1xSOLV+1xSOLV
1xSOLV+1xPQ
1xLPS+1xSOLV
1xLPS+1xPQ
500µm
20µm

## Slide 2
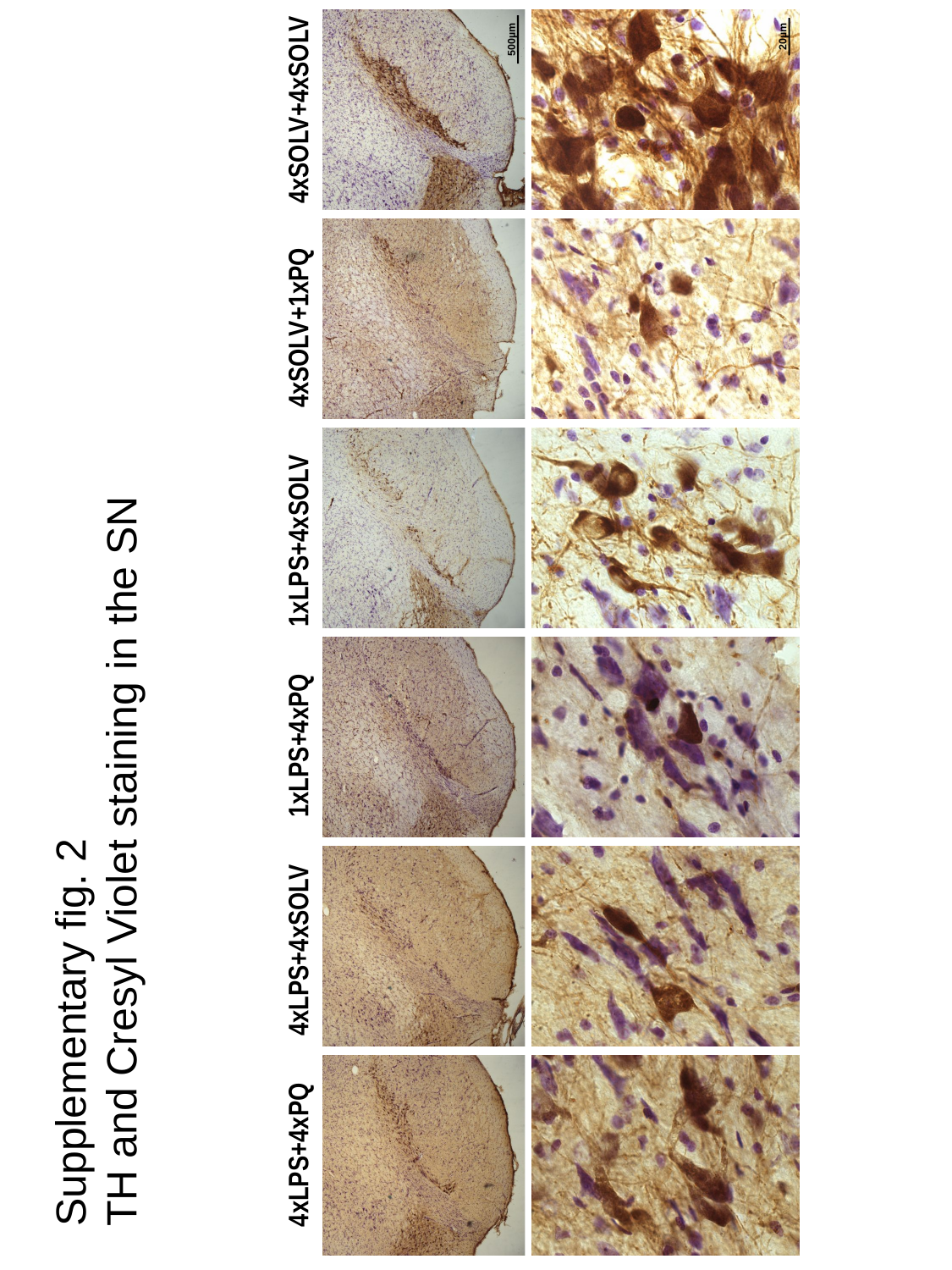

4xSOLV+4xSOLV
4xSOLV+1xPQ
1xLPS+4xSOLV
1xLPS+4xPQ
4xLPS+4xSOLV
4xLPS+4xPQ
20µm
500µm
Supplementary fig. 2
TH and Cresyl Violet staining in the SN
